# The Role of Vitamin C in Cancer Prevention and Therapy: A Literature Review

**DOI:** 10.3390/antiox10121894

**Published:** 2021-11-26

**Authors:** Marcelo Villagran, Jorge Ferreira, Miquel Martorell, Lorena Mardones

**Affiliations:** 1 Biomedical Sciences Research Laboratory, Faculty of Medicine, Universidad Católica de la Santísima Concepción, Concepción 4070129, Chile; marcelo.villagran@ucsc.cl (M.V.); jferreira@ucsc.cl (J.F.); 2 Department of Nutrition and Dietetics, Faculty of Pharmacy and Centre for Healthy Living, University of Concepción, Concepción 4070386, Chile; mmartorell@udec.cl

**Keywords:** ascorbic acid, vitamin C, cancer, clinical trial

## Abstract

Vitamin C is a water-soluble antioxidant associated with the prevention of the common cold and is also a cofactor of hydrolases that participate in the synthesis of collagen and catecholamines, and in the regulation of gene expression. In cancer, vitamin C is associated with prevention, progression, and treatment, due to its general properties or its role as a pro-oxidant at high concentration. This review explores the role of vitamin C in cancer clinical trials and the aspects to consider in future studies, such as plasmatic vitamin C and metabolite excretion recording, and metabolism and transport of vitamin C into cancer cells. The reviewed studies show that vitamin C intake from natural sources can prevent the development of pulmonary and breast cancer, and that vitamin C synergizes with gemcitabine and erlotinib in pancreatic cancer. In vitro assays reveal that vitamin C synergizes with DNA-methyl transferase inhibitors. However, vitamin C was not associated with cancer prevention in a Mendelian randomized study. In conclusion, the role of vitamin C in the prevention and treatment of cancer is still an ongoing area of research. It is necessary that new phase II and III clinical trials be performed to collect stronger evidence of the therapeutic role of vitamin C in cancer.

## 1. Introduction

**Figure 1:**
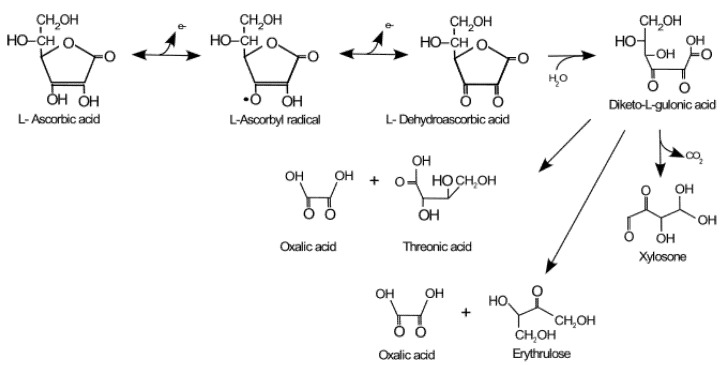
Vitamin C metabolism. In humans, AA is oxidized and degraded into 2–4 carbon products. Translated and modified from Villagran et al. [9].

In 1930, the Hungarian scientists Zilva and Szent-Györgyi isolated and identified the active molecule that prevented scurvy, initially naming it hexuronic acid, a name that was later changed to ascorbic acid (AA) in reference to the deficiency disease, scurvy [1]. AA is the reduced form of vitamin C, while dehydroascorbic acid (DHA) is the oxidized form, and the ascorbyl radical is the intermediate state of oxidation (Figure 1). In water solution, the predominant form is AA, which makes up about 99% of plasmatic vitamin C, while the DHA constitutes only 1%, and ascorbyl radical is negligible (half-life of 10^−5^ s). AA is the main water-soluble, non-enzymatic antioxidant in plasma, where it reaches concentrations of 50–150 µM. When vitamin C plasma levels are below 17 µM and its body reserve is less than 350 mg, scurvy occurs, which is a deficiency disease characterized by bleeding gums, petechiae, and delayed healing [2]. Today, different countries report the prevalence of this deficiency disease between 6% and 12%, with greater susceptibility in four risk groups: the elderly, the indigent, the alcoholic, and the malnourished. There are also strong associations with the socioeconomic and educational levels of the population [3]. However, hypovitaminosis has also been found in cancer and diabetes mellitus patients [4,5]. In myeloid cancer patients, for example, vitamin C hypovitaminosis (<23 μM) was associated with azacitidine chemotherapy [6]. This issue is relevant when cancer patients are treated with vitamin C. Humans are unable to synthesize vitamin C due to the gene mutation of L-gulono-lactone oxidase, an enzyme that catalyzes the final step of AA biosynthesis. Then AA is oxidized in two steps, to ascorbyl radical and DHA, to then be degraded to 2–4 carbon byproducts (Figure 1). In other mammals, vitamin C is synthesized in the liver from glucose. The recommended daily dose (RDD) of AA, according to the Food and Drug Administration (FDA), is 60 mg/day, with a little difference between the sexes and with an extra supplementation in some countries for pregnant and lactating mothers and smokers [5]. The RDD makes it possible to reach a plasma concentration close to 50 μM (0.6 mg/dL) and for the minimum physiological functions of this vitamin to be carried out. The RDD, which is relatively low, reveals that the daily loss is less than 5% of the total body content (1.5 g) and that there is a very efficient recycling system [7]. In species that synthesize AA, there is a high production rate (200 mg/kg/day) without an efficient recycling system, which reveals the relevance of the vitamin C recycling system in maintaining an optimal AA plasmatic level in humans, a species unable to synthetize this vitamin [8]. The RDD of vitamin C can be reached with the daily consumption of three to five fresh fruits or vegetables, mainly citrus fruits, which reach 50 mg/100 g, and also red pepper or kiwi, which contain more than 100 mg/100 g [2].

This review explores the role of vitamin C in available cancer clinical trials and the aspects to consider in ongoing and future studies related to vitamin C (plasmatic and metabolites excretion recording), cancer (i.e., transport and action of vitamin C, type, and state), and the patient (i.e., lifestyle, genetic, age).

## 2. Bioavailability of Vitamin C

**Figure 2:**
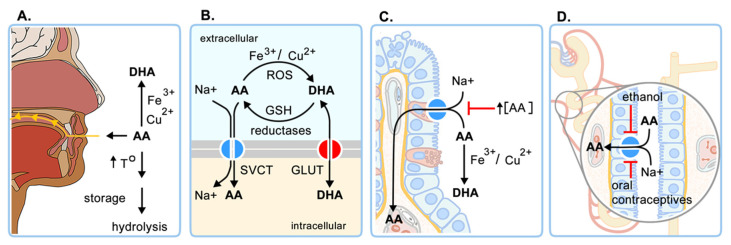
Regulators of vitamin C homeostasis. (**A**) Intake; (**B**) transport and recycling; (**C**) intestinal absorption; (**D**) kidney reabsorption. Effects of different molecules and conditions are also indicated. DHA: dehydroascorbic acid, AA: ascorbic acid, ROS: reactive oxygen species, GSH: reduced glutathione, SVCT: sodium-vitamin C transporter, GLUT: glucose transporter. Translated and modified from Villagrán et al. [9].

Vitamin C homeostasis is controlled mainly by four regulatory processes: (i) intestinal absorption (bioavailability), (ii) accumulation and distribution in tissues, (iii) utilization and recycling, and (iv) renal excretion and reabsorption [1,9] (Figure 2). The intestinal absorption of AA decreases with high intake, due to a decrease in the expression of the sodium ascorbate transporter SVCT1, reducing absorption to 87% for 30 mg, 80% for 100 mg, 72% for 200 mg, 63% for 500 mg, and less than 50% for 1250 mg [10,11]. Unabsorbed vitamin C is eliminated in the feces and the circulating AA is filtered by the kidney, reabsorbed in the proximal tubule by SVCT1, or eliminated in the urine as oxalic acid, AA, or DHA (Figure 1). The absorption of vitamin C and excretion of its metabolites should be considered when high oral doses are administered.

Cells incorporate AA through SVCT1 and SVCT2. SVCT1 is responsible for its intestinal absorption and renal reabsorption and is considered a high-capacity, low-affinity transporter (K_m_ 65–237 µM). On the contrary, SVCT2 is a low-capacity, high-affinity transporter (K_m_ 8–62 µM) with ubiquitous expression and a primary role in vitamin C bioaccumulation [11,12]. DHA is not a substrate of SVCTs, but some isoforms of facilitative glucose transporters (GLUT1, GLUT2, GLUT3, GLUT8, and GLUT10) mediate its transport. The lethal phenotype of SVCT1 and SVCT2 knock out mice, which rules out the relevant participation of GLUTs transporters in vitamin C homeostasis. SVCT1 knock-out has 70% less plasmatic vitamin C and leads to great urinary loss (7 to 10 fold). Meanwhile, SVCT2 knock-out presents intracerebral hemorrhage and serious respiratory failure [8,13]. Despite the relevance of SVCT transporters for survival, in erythrocytes and leukocytes, GLUT1 is responsible for the uptake of DHA, which is then reduced to ascorbate, because those cell types do not express SVCT1/SVCT2. GLUT transporters are also responsible for DHA transport through the blood-brain and blood-testicular barriers [9]. Various enzymes that show DHA reductase activity, such as glutathione S-transferases and thioredoxins, also influence AA concentrations, as they participate in the AA recycling process, which involves the reduction of DHA to AA using GSH or NAPH as a reducing agent (Figure 2) [14].

Another factor that influences vitamin C levels is the presence of polymorphisms in the SVCT1 and SVCT2 genes. It has been reported that seven SVCT1 SNPs are associated with variations in vitamin C levels. Four of them are located in exons 4 and 8, two are in intronic regions, and one is in the 5′ UTR region [11,15]. In the case of SVCT2, various SNPs found in exons 8 and 11 and in introns 2, 3, and 8 have been associated with susceptibility to gastric, colorectal, and neck cancers, and with reduced intracellular AA levels, collagen synthesis, and/or antioxidant defense [16]. SNPs in haptoglobin and glutathione-S-transferase—proteins involved in AA recycling—could also influence intracellular levels of vitamin C [17].

It has been reported that the oral administration of doses greater than 500 mg/day reaches a maximum plasma concentration of 220 μM because its absorption decreases, its excretion increases, and its bioavailability is reduced [10]. On the other hand, intravenous administration of doses greater than 0.4 g/kg reaches stable plasma levels close to 1.5 mM, with a maximum of up to 30 mM. Using pharmacokinetic analysis, Levine et al. predicted that a single dose of 200 mg (from food) could reach a peak of 90 µM in the plasma, meanwhile an intake of 3 g could yield 206 µM. Interestingly, a dose of 1.25 g resulted in a lower concentration peak (187 µM) due to the regulation of vitamin C absorption, excretion, and distribution [10,18]. Hoffer et al. created a formula to determine the plasmatic AA level (AA plasmatic [g/dL] = 3.75 AA doses [g]/weigh [kg]), which could be used in studies where vitamin C is administered [19].

## 3. Physiological Functions of Vitamin C

Among the functions of vitamin C, the most striking are its actions as a neutralizer of free radicals, a regenerator of vitamin E, a cofactor of hydroxylase enzymes that participate in the synthesis of neurotransmitters and collagen, and a regulator of gene expression [20].

Vitamin C intake is associated with the prevention of the common cold [11,21]. However, although the intake of vitamin C reduces the secretion of pro-inflammatory cytokines and the expression of microRNAs associated with inflammation, there is not a confirmed beneficial effect of vitamin C intake for the prevention of colds in the general population [22,23]. In relation to the development of cardiovascular diseases, vitamin C inhibits the oxidation of LDL (low-density lipoprotein), prevents lipid peroxidation, and increases glutathione levels [23,24,25]. On the other hand, at the nervous system level, vitamin C acts as an antioxidant and suppresses the formation of beta-amyloid peptide and glutamate-mediated excitotoxicity [22]. Lately, antecedents have emerged about the participation of vitamin C in the epigenetic regulation of gene expression, as a cofactor in the methylation of cytosine in DNA, and the methylation or deacetylation of lysine and arginine in histones. These actions could be important in the early stages of the development of scurvy or cancer, or could alter the pathogenesis of neurodegenerative diseases [15].

## 4. Anti-Carcinogenic Actions of Vitamin C

**Figure 3:**
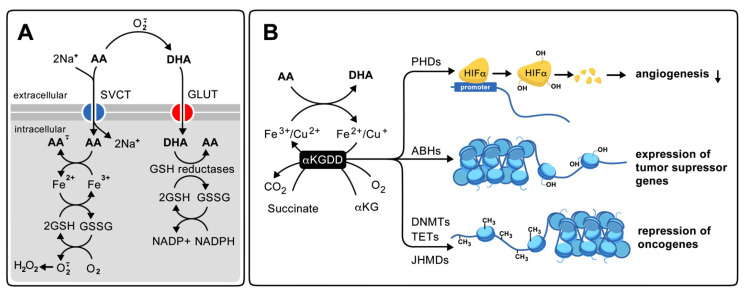
Proposed mechanisms of anti-cancer action of high doses of vitamin C. (**A**): Pro-oxidant; (**B**) regulator of gene expression. DHA: dehydroascorbic acid, AA: ascorbic acid, SVCT: sodium-vitamin c transporter, GLUT: glucose transporter, GSH: reduced glutathione, GSSG: oxidized glutathione, NADP: oxidized nicotinamide dinucleotide phosphate, NADPH: reduced nicotinamide dinucleotide phosphate, αKGDD: α-ketoglutarate-dependent Fe^2+^ dioxygenase enzymes, αKG: α-ketoglutarate, PHD: hypoxia inducible transcription factor alpha prolyl hydroxylases, HIF α: hypoxia inducible transcription factor alpha, ABH: histone Alpha/Beta hydrolase, DNMTs: DNA methytransferases, TET: ten-eleven translocases DNA demethylases, JHMDs: histone demethylases with Jumonji domain. Translated and modified from Villagran et al. [9].

The relationship between vitamin C and cancer is still under study and is associated with antioxidant, pro-oxidant, and gene expression regulator properties (Figure 3) [26]. The effects of vitamin C on cancer progression depend on the route of administration (oral or intravenous), as well as on the expression and compartmentalization of vitamin C transporters in tumor cells. For the expression of transporters of vitamin C in tumor cells, it has been documented that some tumor cells show an increase in the expression of SVCT2 and/or GLUT1 and absorbs more vitamin C than normal cells [26]. The high expression of GLUT1 in tumors constitutes the principle of oncological diagnosis based on the positron emission tomography of ^18^F-fluorodeoxyglucose. For DHA transport by GLUT1, extracellular AA must be oxidized before it can be incorporated into the cell by this transporter. Inside the cell, it is reduced again, which consumes other antioxidants, and contributes to the development of intracellular oxidative stress [27]. On the other hand, a high expression of SVCT2 has been found in various tumors and tumor cell lines derived from breast cancer, mainly at the mitochondrial level [28,29]. Also, Lv et al. found that mega-doses of AA (0.5 mM) kill stem cells from liver cancer with a high expression of SVCT2. The same was observed in xenografts, when mice were given 1.5 g/kg of AA intraperitoneally (equivalent to 1.3 g/kg IV) [30]. Interestingly, it was observed that magnesium ions (5 mM MgCl_2_ or MgSO_4_) synergized with AA to serve as an anti-carcinogen in colorectal cancer cells CT26, human breast cancer cells SK-BR-3 and MCF- treated with AA 1 mM, and in xenograft from these cells in animals that were injected intraperitoneally with 4 g/kg of AA. The authors propose that the effect of magnesium was associated with the activation of SVCT2 through an increase in the maximal rate of transport (V_max_) and oxidative stress [31,32]. However, it is still debated if SVCT2 expression in cancer cells increases the antioxidant or pro-oxidant effect of AA, because in some cases, AA is co-adjunct in radiotherapy or chemotherapy with carboplatin or paclitaxel, but it also was reported that AA protects MCF-7 breast cancer cells from tamoxifen treatment [26,33]. Moreover, DHA acts as a co-adjunct in doxorubicin, methotrexate, and cisplatin chemotherapy, indicative of the relevant role of GLUT as a DHA transporter in cancer cells. Although, vitamin C toxicity was not associated with DHA uptake in non-small-cell lung cancer (NSCLC) and glioblastoma [26,34].

The pro-oxidant action of vitamin C is associated with the reduction of bivalent metals, such as Fe^3+^ and Cu^2+^ by AA, which in the presence of hydrogen peroxide, favors the formation of free radicals through Fenton reaction (Fe^2+^ + H_2_O_2_ = Fe^3+^ + ·OH + OH^−^) and Haber–Weiss (O^2−·^ + H_2_O_2_ + H^+^ = O_2_ + H_2_O + ·OH) (Figure 3) [26,35]. IV AA can produce extracellular H_2_O_2_ of 200 µM/mL, is accumulated in cancer cell mitochondria, and induces caspase-independent autophagy [10,36,37]. Pro-oxidant actions of AA have been observed particularly in Kirsten-Ras (K-ras) and B-raf oncogenes (BRAF) mutant cell lines and were associated with iron toxicity [34,38]. On the other hand, the action of vitamin C as a regulator of gene expression is associated with its antioxidant function, keeping the iron ions in the active site in a reduced state on α-ketoglutarate-dependent Fe^2+^ dioxygenase enzymes (α-KGDDs). Among the αKGDDs regulated by AA are the Jumonji Domain Histone Demethylases (JHDMs), the Ten-Eleven Translocases DNA demethylases (TET), the DNA Methyl Transferases (DNMT), the hypoxia inducible transcription factor alpha Prolyl Hydroxylases D (PHD) and the histone Alpha/Beta Hydrolase (ABH) (Figure 3) [39]. In the DNA, TETs and DNMTs convert 5-methylCytosine (5mC) to 5-hydroxymethylCytosine (5hmC). TETs are recognized as tumor suppressors, while DNMTs were involved in the silencing of tumor suppressor promoters, promoting the methylation of CpG islands. The PHD promotes the degradation of HIFα, reducing the expression of pro-angiogenic genes [39]. Some in vitro studies found that AA, at a physiological concentration (57 μM), increases the inhibitory effect on cell proliferation of suboptimal doses of the DNTM inhibitor 5-azacytidine (5-aza-CdR) in different cancer cell lines, revealing a synergistic effect of AA with this anti-cancerogenic drug. Interestingly, in hematopoietic cell lines (HL60 and HCTT6), the combined treatment had a synergistic effect on the induction of genes that respond to IFN-α and γ, improving viral defense. The mechanism proposed for the combined effect was related to the inhibition of DNMT, the stimulation of TET enzymes, and the increase in the transcription of endogenous retroviruses. The effect of AA could be replicated by DHA [40]. Previously, mutations of DNMT3A or TET2 genes were found in various malignant myeloid disorders [41]. Moreover, the administration of IV vitamin C in patients with hematological malignancies increases the ratio of 5mC:5hmC, indicating the inhibition of DNMT enzymes. In patients with glioblastoma, survival was related to O^6^-methylguanine-DNA methyltransferase gene (MGMT) promoter methylation, underlying the relevant role of AA as an epigenetic regulator in cancer genesis and/or progression [42].

## 5. Vitamin C in Cancer Prevention and Therapy—Studies in Humans

The first studies that evaluated the effect of high doses of vitamin C on cancer were published in the 1970s. Initially, 50 patients with terminal cancer received a combination of IV infusion and oral high-dose vitamin C (5–45 g/day of AA) continuously without chemotherapy. A low number of patients presented beneficial effects in terms of tumor regression and the arrest of its growth (8/50), while three cases presented hemorrhagic lesion and tumor necrosis. Other beneficial effects found were decreased pain at the tumor site or sites of bone metastasis, reduction in ascites, jaundice, hematuria, and the blood sedimentation rate [43]. The authors did not include controls in their study, and they used different treatments (oral and IV), dose combinations, routes of administration, and treatment times (up to 2.5 years for patients in which the treatment was well tolerated). They also reported side effects, such as gravitational edema and dyspeptic symptoms. They replicated the study in 100 patients, comparing them with 1000 control patients [44,45]. In this case, an increase of up to 5.5 times in viability was observed, with 22 patients presenting over 3.5 years in survival, when compared with a control case with a similar pathological condition (type of tumor, time of diagnosis of tumor, and time of diagnosis of terminal illness). The authors included 50 cases of the previous study, pointing out that the dose administered was usually 10 mg/kg by IV daily. This study did not include randomization of the patients, which can produce bias in data analysis. Similarly, but randomized, the subsequent study did not observe beneficial effects of this vitamin on patient survival or symptoms, although in these studies, vitamin C was administered orally (10 g/day of AA) [46]. The studies did not consider the effect of the route of administration of vitamin C and its plasma levels. On the other hand, the studies included terminally ill patients with different types of cancer, where complications and immunocompetence can influence responses to vitamin C treatment. Nowadays, some controversy persists about the effectiveness of the administration of vitamin C during the progression of cancer, as a co-adjunct to chemotherapy, or in reducing its adverse effects, but there is more information in support of the effectiveness, including cellular and molecular mechanisms involved [47].

### 5.1. Clinical Trials

Since the 1970s, countless studies have been carried out to analyze the effects of the administration of vitamin C on cancer prevention and progression, with 90 registers in the databases of clinical trials of the European Union and the North American government. There are only three records on the EU, and only one with available results. Meanwhile, in the U.S. database, of the 87 registers, only 18 have available results. Several studies were discontinued due to the development of adverse effects, such as anemia, neutropenia, hypertension, embolism, and thrombocytopenia. The effects were associated with the disease, the chemotherapy, or the AA treatment.

In the clinical trials already completed, the study NCT0272428 analyzed the effect of antioxidant intake on the prevalence of cancer in 12,741 adults between 35 and 60 years of age using a randomized, double-blind, and placebo-controlled model; this study was part of the SU.VI.MAX study (Supplementation in Vitamines et Mineraux Antioxydants), which is one of the few registered phase III studies) [48]. A total of 6364 subjects received a daily dose of 120 mg of vitamin C, 30 mg of vitamin E, 100 UI of beta carotene, 6 mg of selenium, and 100 mg of zinc, while 6,377 subjects constituted the control group. No differences in cancer incidence were observed between the placebo and control groups for 7.5 years of recording, but, when grouping by sex, there was a reduction of cancer incidence in men (risk 0.69 vs. 1.06) and death incidence (relative risk 0.63 vs. 1.03) (Table 1). The authors conclude that the positive effects of the continuous intake of low doses of antioxidants in men could be due to a lower baseline status of antioxidants in general, especially of beta carotene. On the other hand, another randomized interventional phase III study (NCT00270647) analyzed the development of cancer and other diseases in 14,641 men older than 50 years who received a combination of vitamins. The analysis lasted for 8 years and did not show a preventive effect of the administration of 500 mg of vitamin C/day and 400 IU of vitamin E/day alone or in combination with beta carotene (50 mg of lurotin/day), on the prevention of prostate cancer, nor in the incidence of all types of cancer during the 14-year follow up [49,50]. However, in an 11-year follow-up study, it was found that the administration of the multivitamin reduced the re-incidence of cancer (R 0.73, *p* = 0.02) [51]. The Eudra-CT2008-008692-33/NCT01080352 corresponds to a noncomparative phase II study that proposed to recruit 80 Danish men with castration-resistant prostate cancer and evaluates the effect of IV AA. The study included only 20 patients and did not find beneficial effects of treatment with vitamin C, even though there were various adverse effects that required hospitalization, such as hypertension and embolism [52]. AA treatment included increasing IV doses for 12 weeks (5, 30, and 60 g, infusion of 1 g/min) followed by 14 weeks of oral AA (500 mg). An AA plasma concentration of 90 μM was achieved at the end of the complete treatment, with a peak of 20 mM [53]. No reduction in plasma levels of prostate antigen (PSA), oxidative damage (excretion of 8-oxoguanidine), and bone metastasis was observed, implicating that it is not recommended to use high doses of IV vitamin C in patients with prostate cancer.

Among the clinical trials in which the anti-carcinogenic effect of AA was analyzed, the study NCT0105062 treated 14 patients with different malignancies with 1.5 g/kg of AA IV, three times a week. Urinary oxalate and plasmatic AA were monitored during the treatment [54]. Moreover, the hypovitaminosis of cancer patients was normalized by 0.6 g/kg of AA before chemotherapy. The result showed no clear anti-cancer effect of AA, because improvement of symptomatology and stabilization of cancer progression was observed in only 6 patients (Table 1). It was concluded that new research on the carcinogenic effect of IV AA needed to be undertaken, and that it was important to carry-out the analysis using an individual case-oriented strategy.

The clinical trial NCT00006021 studied the effect of a combination of arsenic trioxide (25 mg/kg/day) and IV AA (1 g/day) for 25 days with a week diphase in 22 patients with refractory or recurrent multiple myeloma and found that AA plasmatic levels reached 180 μM at 1 h, which was associated with glutathione depletion [55]. The authors found a decrease in protein M and detention of cancer progression in few patients (Table 1). The anti-carcinogenic effect of arsenic trioxide has previously been associated with apoptosis and mitochondrial oxidative stress, and the effect of AA or GSH depletion on its pharmacokinetic and possible resistance should be explored [59].

There are four small clinical trials that studied the effect of IV AA on pancreatic cancer. The NCT01515046/NCT01049880 studied the effect of vitamin C–gemcitabine combined treatment in 9 patients with adenocarcinoma grade IV. The gemcitabine was administrated in a scheme of 2 cycles of 1000 mg/m^2^ for 3 weeks plus a week off. AA was given in escalated doses weekly, beginning with two doses of 15 g IV weekly, continuing with escalating doses in 25 g increments until a 20 mM plasma level of vitamin C was reached. The study found a plasmatic AA level duplication (83 vs. 44 μM, *p* < 0.001) and a decrease in plasmatic F2 isoprostanes, but no changes in the ascorbyl radical level or GSH oxidation. In addition, lower weight loss and an overall survival of 12 months were observed, compared with weak side effects, such as diarrhea and dry mouth. It was concluded that pharmacological doses of vitamin C were well-tolerated and did not induce systemic oxidative stress (Table 1) [56]. The study continued with a phase II clinical trial (NCT2905578) still in development. In a related phase I clinical trial (NCT02248584), changes in plasmatic 15-F2t isoprostanes were not found in 60 patients with skin cancer supplemented with vitamin C, E, and Zn [60]. The phase I clinical trial study NCT01364805 included 14 patients with advanced or metastatic pancreatic cancer and showed a decrease in metastasis and cancer cell proliferation (Table 1). In this study, AA (100 g, 3 times weekly) was also administered in combination with standard doses of gemcitabine. In an additional study, using human and murine pancreatic cell lines, NAD+ and ATP depletion, and α-tubulin acetylation, together with inhibition of epithelial-mesenchymal progression was observed in response to 20 mM AA. Energetic unbalance was prevented by catalase, which involves H_2_O_2_ in AA anti-carcinogenic actions. Results were also observed in a xenograft mice model treated with 4 g/kg of AA daily, alone or in combination with gemcitabine (40 mg/kg, 3 times weeks) for 45 days, using the same cell tumoral lines [57]. The study NCT00954525 included 14 patients with grade IV metastatic ductal pancreatic adenocarcinoma treated with gemcitabine (1000 mg/m^2^ weekly for 7 weeks) and erlotinib (100 g, daily for 8 weeks). AA was administered in escalated doses (50, 75, and 100 g, 3 times weekly, for 8 weeks). IV AA had no effect in chemotherapy, nor did it lead to any side effects, but the tumor size decreased (Table 1). In addition, the authors reported that plasmatic AA reached 30 mM [58].

Between the two EU records still in development, there is a phase II randomized controlled and clinical study (Eudra-CT 2019-004074-25). This study proposes to analyze the protective effect of the combination of two antibiotics (doxycycline and azithromycin) with vitamin C on 90 women with early breast cancer. As a background, the authors report that the combination of these two antibiotics in sub-antimicrobial doses (1 μM) with high doses of vitamin C (250 μM) prevent the spread of cancer stem cells in breast cancer cell lines [37]. In vitro triple treatment reduced the mitochondrial metabolism, ATP levels, cellular glycolytic reserve, and glycolysis. The authors propose that doxycycline inhibits mitochondrial translation, vitamin C acts as pro-oxidant and induces mitochondrial biogenesis, and azithromycin stimulates mitophagy. Mitochondrial vitamin C accumulation occurs with SVCT2 expression in breast cancer cells [61]. The other study in the EU database that is still in development is an observational study (Eudra-CT2018-004135-77/NCT02931942). The researchers propose to study the levels of AA in 150 patients treated with intensive (leukemias and other hematological malignancies) and medium (lung and colon cancer) anticancer therapy, complemented with the NK cell count. Evidence in this regard indicates that chemotherapy causes a decrease in AA levels, and that in vitro AA (50 μg/mL) promotes NK-cell differentiation from early T/NK-cell progenitors [62].

We only discussed clinical trials with available results; however, most of them are phase I assays with few participants and single cases, which made it difficult to obtain a general conclusion. It is evident that the more recent studies complement clinical data with in vitro and experimental studies and follow plasmatic AA levels, but only one followed a byproduct compound (oxalic acid). The diversity of results suggests that the response of a cancer patient to vitamin C treatment depends on the disease (cancer type, mutation involved, and state) and on AA (doses, frequency, source of administration). We must wait for more data to become available from the several clinical trials still in progress to obtain more conclusions about vitamin C’s effect on cancer prevention or treatment.

### 5.2. Meta-Analysis and Mendelian Randomization Studies

We found 13 meta-analyses that studied the relationship between vitamin C and cancer. Of them, four studied the relation between vitamin C intake and the relative risk of cancer development in general. A 2015 study developed by Lee et al. included seven articles that encompass 62,619 participants [63]. The vitamin C treatment was performed for 5 to 10 years with oral doses between 120 and 500 mg per day of vitamin C alone or in combination with other vitamins (Table 2). The results revealed no beneficial effects of the supplementation on decreasing cancer incidence (relative risk RR 1.00, 95% confidence interval CI, 0.95–1.05). The authors pointed out that the studies analyzed only included adults between 50 and 60 years who used commercial vitamin C; for that reason, the date cannot be extra-poled to younger people or to the intake of natural sources of the vitamin. In 2018, Aune et al. analyzed 69 published prospective studies where the effect of the intake of vitamin C, carotenoids, and α-tocopherol from natural sources on cancer incidence was evaluated. The meta-analyses considered 16 randomized studies, involved 32,601 participants, and found a statistically significant reduction of total cancer incidence (RR 0.93, 95% CI 0.87–0.99) (Table 2). In addition, the study also found negative correlation between blood vitamin C and cancer development in the group with the lowest vitamin C intake (RR 0.74, 95% CI 0.66–0.82) [64]. The authors associated this last finding with the saturation of blood with vitamin C. Errors due to confounding factors, such as physical activity, body weight, smoking, alcohol intake, and/or red meat consumption, were not analyzed. A 2019 meta-analysis performed by van Gorkom et al. analyzed the relationship between vitamin C levels and cancer development in general [65]. The study included 19 publications between 1974 and 2018, of which only four were randomized studies. Vitamin C was administered orally in three studies, orally and intravenously in eight, and in parallel with anticancer drugs in seven studies. The doses of vitamin C administered were in the range of 30 mg to 50 g per day for oral administration and 50 mg to 150 g per day for intravenous administration, following different protocols (continuous, alternate, or escalated doses) and durations of treatment (10 days to 12 months) (Table 2). Better effects were observed after IV administration and no major side effects were reported in any case, both independently of doses and by the manner of administration. It was concluded that there was no evidence of beneficial effects from the administration of vitamin C on survival, quality of life, or clinical status of patients, but it was also stated that the quality of the studies reviewed was low (risk of detection of bias) and that the groups were very diverse. It is, therefore, clear that effect of vitamin C on incidence and progression of cancer in general is still a matter of controversy due to the complex disparities in the studies analyzed.

In relation to a meta-analysis performed on cancer types, we comment on three meta-analyses related to prostate cancer incidence. Stratton et al. analyzed 14 studies, encompassing 4 randomized clinical trials, 8 cohort studies, and 2 case-control studies, which included a total of 265,932 subjects. The authors found no association between multivitamin intake and development of prostate cancer [66] (Table 2). Similarly, Jiang et al. reported no effect of vitamin C supplementation intake in prostate cancer incidence [67]. This study analyzed a total of 9 randomized clinical trials that included 165,056 subjects in all, but only in 2 studies was vitamin C administrated. In those two studies, supplementation showed a beneficial effect (RR 0.98, 95% CI 0.91–1.06) (Table 2). The meta-analyses of Bai et al., which included 18 studies and a total of 103,658 subjects, found an inverse correlation between vitamin C intake and development of prostate cancer (RR: 0.89, 95%CI: 0.83–0.94) [68] (Table 2). Similar results were obtained when the data was grouped by cohorts or case-study. The study included patients between 40 and 86 years, a dosage of vitamin C from food sources between 70 and 250 mg per day and a follow-up between 3 and 30 years. Interestingly, the authors also reported an inverse dose–response relationship between vitamin C intake and prostate cancer risk, obtaining 21% less risk with the higher vitamin C doses intake. In general, there was no conclusive evidence about the beneficial effect of vitamin C intake in prostate cancer development, but this could be associated with the heterogenicity of studies included, the different types of analysis performed, time of treatment, dose used, and source of vitamin C.

There are two meta-analyses on lung cancer development. In 2006, Cho et al. analyzed 8 published randomized clinical trials that included 430,281 subjects [69]. The vitamin intake from natural or artificial sources was between 70 and 200 mg per day and the follow up was from 6 to 16 years. The data showed a 28% decrease in lung cancer incidence between the highest and the lowest quintile in an age-adjusted analysis within participants with food-only vitamin C intake (RR 0,86, 95%CI 5 0.76–0.99) (Table 2). However, this association disappears when β-cryptoxanthin content is considered. The study found no effect from taking other vitamins, sex, smoke status, or lung cancer type. Another meta-analysis related to lung cancer was performed in 2014 by Luo et al., who analyzed 21 studies involving 8938 patients between 20 and 89 years [70]. The authors found that the risk of lung cancer decreased by 7% for every 100 mg/day increases in the intake of vitamin C (RR 0.93, 95%CI 5 0.88–0.98), with a similar result obtained under different classifications: prospective studies (7), U.S. citizens (17), men (8), adenocarcinoma patients (3), and squamous cell carcinoma patients (3) (Table 2).

There are four meta-analyses related to vitamin C intake and breast cancer incidence. In a meta-analysis carry-out in 2000, Gandini et al. found a protective effect of vitamin C consumption (RR 0.80, 95% CI, 0.68–0.95) [71]. The vitamin C intake was between 50 and 400 mg per day in the 9 studies analyzed, which included a total of 278,847 subjects (10,740 treated and 268,107 controls) (Table 2). The authors emphasized the heterogenicity of the analysis and the design of the different studies, including obtention of diet information with different food frequency questionnaires. Fulan et al. performed a meta-analysis that included 34 case-control studies and 475,234 subjects. They found that dietary vitamin C alone or plus a supplement of vitamin C reduces breast cancer incidence by 15 and 23%, respectively (OR 0.85, 95% CI: 0.77–0.93, *p* < 0.01 and OR 0.77, 95% CI: 0.68–0.87; *p* < 0.01) (Table 2) [72]. The vitamin C intake in the included studies was between 100 and 300 mg/day. Another study from the same authors, published in 2015, found an association between breast cancer development and plasmatic vitamin C (−2.51 μM [95% CI −4.00, −1.02, *p* = 0.001]) [73] (Table 2). It included a total of eight case-control studies related with vitamin C, and the heterogenicity was reduced when only pre- and post-menopausal women were included, while the protective effect was maintained. A meta-analysis of Harris et al. included 13 prospective observational studies with a total of 17,696 women treated with dietary (six studies) or supplemented (seven studies) vitamin C intake [27]. The results suggested that post-diagnostic vitamin C intake was associated with a reduced risk of mortality (RR 0.85, 95% CI, 0.74–0.99). Several of the studies were adjusted by body mass index, clinical characteristics of the tumor, and energy intake. In 2020, Zhang et al. published a meta-analysis that included 69 studies and 17,067 women [74]. The results revealed no association between vitamin C intake and breast cancer risk (RR 0.85, 95% CI, 0.74–0.99), but there was significant association when only case-control studies were included (RR 0.74; 95% CI, 0.65–0.84; *p* < 0.001) and when dietary intake studies were included (RR 0.89; 95% CI, 0.82–0.96; *p* = 0.004) (Table 2).

A single Mendelian randomization (MR) study on the relationship between circulating vitamin C levels and the risk of developing cancer was published in 2021 [75]. This MR study statistically analyzed the influence of nine gene variants on circulating vitamin C levels, as well as the causal effect of vitamin C levels on the development of lung, prostate, breast, colon, and rectal cancer. In total, data from 870,984 subjects were included for the MR analyses and data from 52,018 subjects for the reverse MR analyses. For reverse MR, data from four consortia with available GWAS (genome-wide association study) data were included: the Fenland study, the European Prospective Investigation into Cancer and Nutrition (EPIC)-InterAct study, the EPIC Norfolk study, and the EPIC-CVD study. For MR studies, data from the UK Biobank International Lung Cancer Consortium (ILCCO), the Prostate Cancer Association Group to Investigate Cancer-Associated Alterations in the Genome (PRACTICAL) consortium, and the Breast Cancer Association Consortium (BCAC) were included. In addition, the researchers conducted a meta-analysis of published data from prospective cohort studies and randomized clinical trials, analyzing data from a total of 1,992,894 participants. The meta-analysis of all the data available reveals that there was only a negative correlation for lung cancer with vitamin C levels (rg = −0.43, *p* = 0.01), results that were confirmed in the reverse MR. However, the effect disappeared when SNPs with potential pleiotropy were eliminated, and data were adjusted for tobacco consumption. On the other hand, a negative correlation between vitamin C levels and development of breast cancer was found in the UK Biobank data analysis (OR 1.34, *p* < 0.001). After analyzing the available data using six different MR methods, the authors concluded that there was no association between circulating levels of vitamin C and the development of the five types of cancers studied. Because previous meta-analysis studies found preventive effects of vitamin C intake from natural sources, more than from supplements, the authors postulated that antioxidants in fruits and vegetables, e.g., polyphenols, can influence the preventive effect of vitamin C.

Although meta-analysis studies have higher statistical power than isolated studies, their analyses of the data are often very complex, mainly due to the variety of the studies included, population characteristics, vitamin C doses, sources and routes of administration, and the inclusion or exclusion of confounding factors. The data reviewed here show that vitamin C has a protective effect in lung and breast cancer, particularly when it is ingested through the patient’s diet. This information is indicative of a combined effect of vitamin C with other antioxidants present in food or a healthy diet and can be generally beneficial. It is important to perform other randomized Mendelian studies associating vitamin C intake with cancer development and mortality.

## 6. Conclusions

In conclusion, the relationship between vitamin C and cancer is still under study. The differences in results depend not only on issues related with AA (doses, routes of administration, plasmatic levels, metabolites recording, and source), but also on cancer characteristics (presence of specific mutations, cancer type and grade, and conventional anti-cancer therapy received) and patient characteristics (diet, behaviors, renal dysfunction, genetics, and SNPs in SVCT transporters). This allows the monitoring doses to be effective, and then associates them with an anti-cancer mechanism involved. Meta-analysis studies show that AA from foods prevents breast and lung cancer; however, the Mendelian randomized study did not find any association between plasmatic vitamin C and cancer incidence. Clinical trials with IV AA as a co-adjuvant in radiotherapy or chemotherapies with gemcitabine, erlotinib, and DNTM inhibitors are assays in development with promising results. It is expected that new studies will reveal more about the mechanisms of the anti-cancer action of vitamin C, with new, well-structured phase II and III clinical trials performed to confirm its proposed beneficial effect on cancer prevention and treatment.

## Figures and Tables

**Table 1:** Clinical Trials.

Code	N° Subjects	Cancer Type	Finding	Ref.
NCT0272428	12,741	all	↓incidence in men (7,5 yr.)	Hercber et al. [48]
NCT00270647	14,641	all	↓re-incidence in men (11 yr.)	Gaziano et al. [49]
Eudra-CT2019-004074-25/ NCT01080352	20	prostate	Media-severe side effects	Nielsen et al. [52]
NCT0105062	14	all	↓symptoms/progression (43%)	Hoffer et al. [54]
NCT00006021	22	myeloma	↓progression (50%)	Bahlis et al. [55]
NCT01515046/ NCT01049880	9	pancreas	↑survival (12 mo.)	Welsh et al. [56]
NCT01364805	14	pancreas	↓metastasis and cell proliferation	Polireddy et al. [57]
NCT00954525	14	pancreas	↓tumor size	Monti et al. [58]

**Table 2:** Meta-analysis studies.

N° Subjects	Cancer Type	Finding	Ref.
62,619	all	No effect in incidence	Lee et al. [63]
32,631	all	↓incidence	Aune et al. [64]
5074	all	No effect in incidence	Van Gorkom et al. [65]
265,932	prostate	No effect in incidence	Stratton et al. [66]
165,056	prostate	No effect in incidence	Jiang et al. [67]
103,658	prostate	↓incidence	Bai et al. [68]
430,281	lung	No effect in incidence	Cho et al. [69]
8938	lung	↓incidence	Lou et al. [70]
278,847 475,234	breast breast	↓incidence No effect in incidence	Gandini et al. [71] Fulan et al. [72]
908	breast	↓incidence	Hu et al. [73]
17,296 17,067	breast breast	↓mortality No effect in mortality	Harris et al. [27] Zhang et al. [74]
